# Development of composite separators by coating hydrochloric acid-treated halloysite nanotubes on polypropylene separators for lithium-ion batteries†

**DOI:** 10.1039/d4ra02164a

**Published:** 2024-05-24

**Authors:** Qinting Deng, Xiuyun Chuan, Yupeng Zhao, Fangfang Liu, Shizhi Huang, Jianyang Wu

**Affiliations:** a Key Laboratory of Orogen Belts and Crustal Evolution, School of Earth and Space Sciences, Peking University Beijing 100871 China xychuan@pku.edu.cn; b College of Chemistry and Molecular Engineering, Peking University Beijing 100871 China

## Abstract

In this study, polypropylene/halloysite nanotube (PP/HNT) composite separators were prepared by coating HNTs treated with hydrochloric acid (HCl) of different concentrations on both sides of a PP separator. The effect of HNTs treated with hydrochloric acid (HCl) of different concentrations on the properties of PP/HNT composite separators was investigated. The results indicate that the PP/HNT composite separator exhibits higher electrolyte uptake and wettability than a commercial PP separator, resulting in a better electrochemical performance in Li/LiFePO_4_ cells. In particular, the PP/HNTs-1.2 M composite separator with HNTs treated with 1.2 M HCl exhibits the highest electrolyte uptake (384%) and ionic conductivity (1.03 mS cm^−1^). The cells assembled with a PP/HNTs-1.2 M composite separator deliver discharge capacities of 166 mA h g^−1^ (0.5 C) and 131 mA h g^−1^ (3 C) with attractive cycling performance (87.6% capacity retention after 100 cycles). HNTs treated with HCl of appropriate concentrations can significantly improve the properties of PP/HNT composite separators for application in lithium-ion batteries.

## Abstract 2

(1) HNTs treated with HCl of different concentrations were coated on both sides of PP separators to prepare PP/HNT composite separators. (2) The PP/HNT composite separators exhibited improved physical and electrochemical properties.

## Graphical abstract

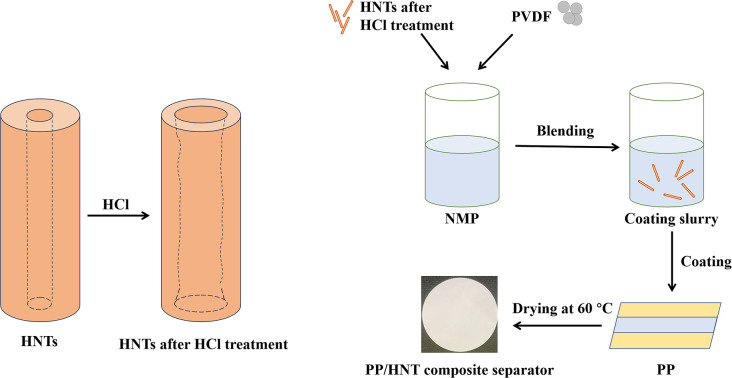

## 1. Introduction

Lithium-ion batteries (LIBs) have been widely used in new energy vehicles and electronic equipment owing to their high energy density, long cycle life, low self-discharge rate and no memory effect.^1–4^ As a key component of batteries, separators not only isolate anodes and cathodes to avoid internal short circuiting, but also allow the transport of lithium ions in liquid electrolytes throughout the porous structure.^5,6^ Nowadays, commercial polyolefin separators such as polyethylene (PE), polypropylene (PP) and their compounds are commonly used in LIBs owing to their excellent mechanical strength, good electrochemical stability and reasonable cost.^7,8^ However, their poor thermal stability causes separators to shrink easily at higher temperatures, which causes fire and explosion accidents. Besides, the low electrolyte wettability limits the development of high-performance batteries.^9,10^

Considerable studies have been conducted to overcome these limitations. Natural minerals have the advantages of unique nano-pore structures, excellent thermal stability, low costs and environmentally friendly nature. Therefore, many natural minerals are used as inorganic fillers to improve the electrolyte wettability and thermal stability of separators.^11–13^ For example, Song *et al.*^14^ prepared a SA (sodium alginate)/ATP (attapulgite) separator *via* a phase inversion method, which exhibited high thermal stability, attractive cycling stability and rate capability. Xiao *et al.*^15^ prepared a shutdown functional separator by coating high-density polyethylene (HDPE) wax@boehmite (AO) on a PE separator. The HDPE@AO coating layer could be preferentially melted at about 130 °C, which could block the pores of PE and shut down the transport of lithium ions. Carter *et al.*^16^ prepared a flame-retardant vermiculite-coated PP separator, which released less exothermic energy during thermal runaway and enhanced battery safety.

Halloysite nanotubes (HNTs) are among the natural minerals with a special hollow tube structure and unique charge characteristics. The positively charged inner lumen contains aluminol (Al–OH) groups and the negatively charged outer surface contains siloxane (Si–O–Si) groups.^17–19^ Hence, HNTs have attracted widespread interest for separator modification. Xie *et al.*^20^ coated HNTs on both sides of a PP separator to prepare a HNTs@PP separator, and the three-dimensional space structures and one-dimensional channels of HNTs promoted the transport of lithium ions and improved the cycle and rate performance. To test the universality of the action of HNTs, Huang *et al.*^21^ developed a highly thermally stable separator by coating HNTs on both sides of commercial papers or waste newspaper. Xu *et al.*^22^ prepared HNT/PVDF composite separators by a phase inversion method, which showed excellent electrolyte uptake and low interfacial impedance.

The acid treatment of HNTs can selectively etch alumina sheets within their lumen and controllably enlarge the lumen diameter and specific surface area.^23–25^ Abdullayev *et al.*^26^ treated HNTs with sulphuric acid and the specific surface area of the tubes increased over 6 times by selective etching of 60% alumina within the tube lumens. Garcia-Garcia *et al.*^27^ studied the effect of different acids on the treatment of HNTs, and the results indicated that the aluminium reduction and acid strength were positively correlated. Wang *et al.*^28^ investigated the effect of HCl of different concentrations on HNTs, which indicated that the BET surface area and pore volume of acid-treated HNTs increased with the increase in HCl concentration. Nevertheless, to our knowledge, an in-depth study of the effects of HNTs treated with HCl of different concentration on separators in LIBs has not yet been reported.

Therefore, in this study, we etched HNTs with HCl of different concentrations and coated them on both sides of the PP separator. Moreover, the effect of HNTs treated with HCl of different concentrations on the physical and electrochemical properties of separators for LIBs was investigated.

## 2. Experimental

### 2.1. Materials

Natural halloysite nanotubes (HNTs) were purchased from Hunan Xianglei Kaolin Industrial Co., Ltd. Hydrochloric acid (HCl, 36%) and *N*-methylpyrrolidone (NMP, 99.89%) were obtained from Xilong Science Co., Ltd. Polyvinylidene fluoride (PVDF, 99.95%) and acetylene black (99.95%) were provided by Cyber Electrochemical Materials Network. A commercial polypropylene (PP) separator (Celgard 2500) was purchased from Celgard Company (USA). A LiFePO_4_ powder was purchased from Pulead Technology Industry Co., Ltd. A lithium plate was obtained from China Energy Lithium Co., Ltd. An electrolyte solution (1 M lithium bis(trifluoromethanesulfonyl)imide (LiTFSI) solution in 1,2-dimethoxyethane (DME) and 1,3-dioxolane (DOL) in a volume ratio of 1 : 1 + 2% LiNO_3_) was supplied by Shanghai Songjing New Energy Technology Co., Ltd.

### 2.2. Preparation of HNTs treated with HCl

First, 1.3 g purified HNTs were dispersed in 30 mL HCl solution at concentrations of 0.4, 0.8, 1.2 and 4 M, respectively. Then the dispersion was poured into a hydrothermal reactor and heated in an oven at 180 °C for 10 h. The obtained precipitate was washed, filtered and dried to obtain HNTs treated with HCl. The HNTs purified and treated with 0.4, 0.8, 1.2 and 4 M HCl solution were named HNTs-0 M, HNTs-0.4 M, HNTs-0.8 M, HNTs-1.2 M and HNTs-4 M, respectively. The mass reduction rates of different HNTs are provided in Table S1.†

### 2.3. Preparation of PP/HNT composite separators

**Fig. 1:**
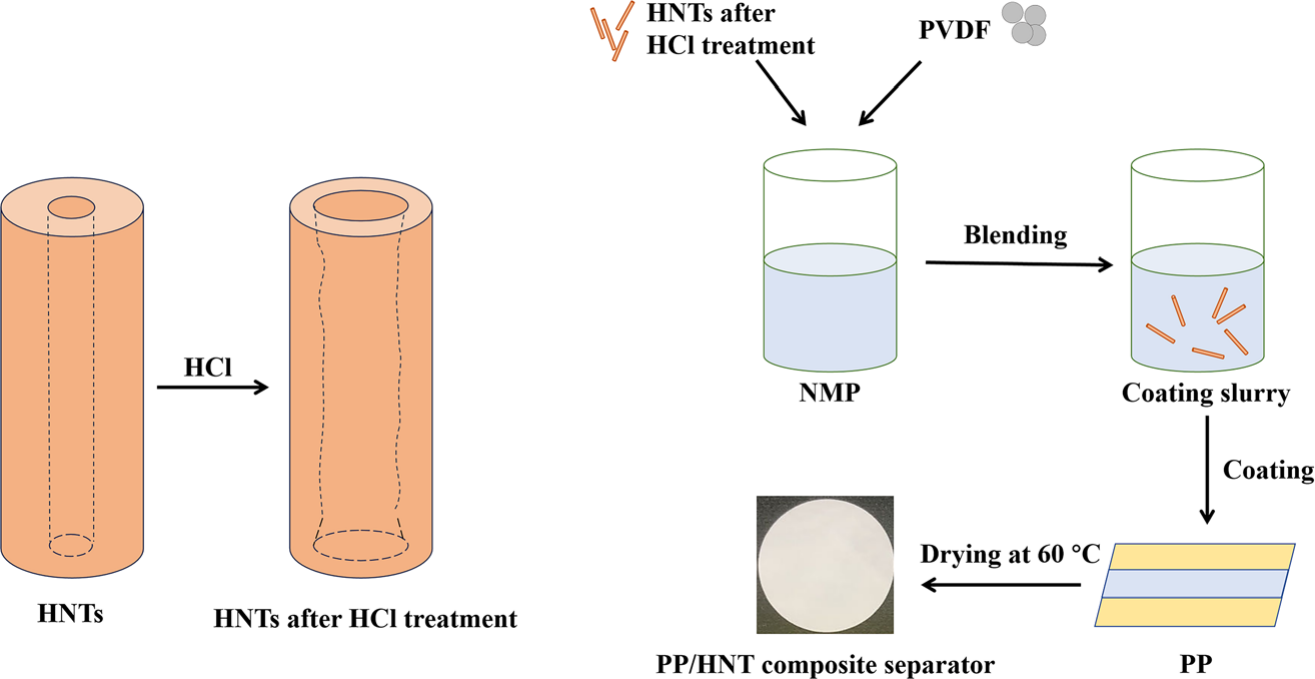
Preparation process of PP/HNT composite separators.

The preparation method was referred from a previous study reported in the literature.^20^ PP/HNT composite separators were prepared *via* a simple coating process (Fig. 1). The coating substrate was the PP separator (Celgard 2500, thickness: 25 μm). Typically, the obtained HNTs and PVDF were added into an NMP solution in a mass ratio of 95 : 5. After stirring for 12 h, the mixed solution was coated on both sides of the PP separator using an automatic coating machine. After coating, the PP/HNT composite separators were dried in a vacuum drying oven at 60 °C for 12 h. The obtained composite separators were named PP/HNTs-0 M, PP/HNTs-0.4 M, PP/HNTs-0.8 M, PP/HNTs-1.2 M and PP/HNTs-4 M respectively, and their thickness was controlled at 35 μm. The loading of HNTs on the PP/HNT composite separator was about 1.0 mg cm^−2^.

### 2.4. Materials characterization

The surface morphologies of the PP and PP/HNT composite separators were characterized using a field emission environmental scanning electron microscope (ESEM, Quanta 200F, FEI). The hollow tubular structure of the HNTs was characterized using a field emission transmission electron microscope (TEM, Tecnai F30, FEI). The pore structure of the HNTs was characterized using an accelerated surface area and porosimetry system (ASAP 2020, Micrometer). Fourier transform infrared (FTIR) spectra were recorded using a Fourier transform infrared spectrometer (*Nicolet iS50*, ThermoFisher). X-ray diffraction patterns were recorded using a powder X-ray diffractometer (X-Pert3 Powder, PANalytical). The contact angles (CAs) of the PP and PP/HNT composite separators were measured using an optical contact angle measuring instrument (DSA30, KRUSS).

The electrolyte uptake (EU) of the separators was measured by immersing them in the electrolyte for 2 h, taking out of the solution and gently wiping off the electrolyte on the surface of the separators with filter paper. It was calculated using the following formula:1
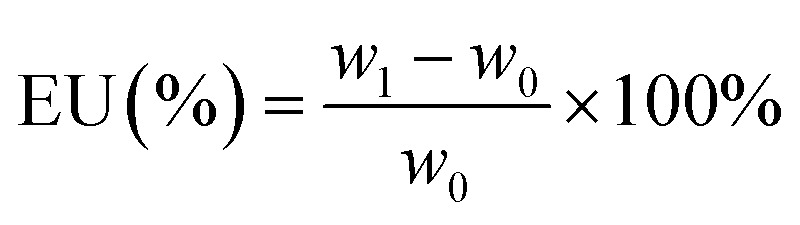
where *w*_0_ and *w*_1_ are the masses of the dry and wet separators, respectively.

The porosity of the separators was measured by immersing them in *n*-butanol for 2 h, taking out of the solution, and gently wiping off *n*-butanol on the surface of the separators with filter paper. The porosity was calculated using the following formula:2
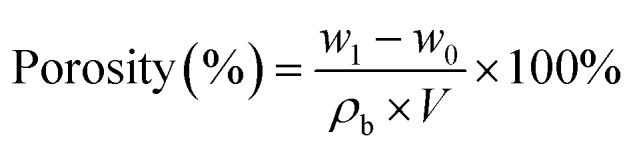
where *w*_0_ and *w*_1_ are the masses of the dry and wet separators, respectively, *ρ*_b_ is the density of *n*-butanol, and *V* is the volume of separators.

The thermal stability of separators was tested by heating them at 170 °C for 0.5 h. The mechanical properties of the PP and PP/HNT composite separators were analyzed using a microcomputer control electronic universal testing machine (UTM2503X, Shenzhen Sansi Zongheng Technology Co., Ltd).

### 2.5. Electrochemical measurements

To evaluate the electrochemical performance, 2032-type lithium coin cells were assembled by employing separators containing PP, PP/HNTs-0 M, PP/HNTs-0.4 M, PP/HNTs-0.8 M, PP/HNTs-1.2 M and PP/HNTs-4 M, respectively.

To test the ionic conductivity of the separator, it was sandwiched between two stainless steels (SS) to form an SS|separator|SS cell. The cell was tested using an AC impedance spectrometer on an electrochemical workstation (CHI660C, Shanghai Chenhua Co., Ltd) in the frequency range from 10^5^ Hz to 0.01 Hz with a scan amplitude of 0.005 V. The ionic conductivity was calculated using the following formula:3
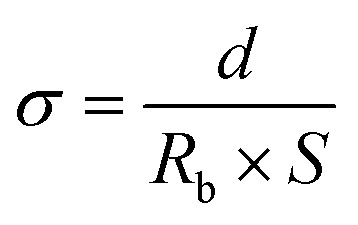
where *σ* is the ionic conductivity, *d* is the thickness of the separator, *R*_b_ is the bulk resistance, and *S* is the surface area of the separator.

To study the effect of separator on battery performance, electrochemical impedance spectroscopy (EIS) was performed at a frequency of 10^5^ Hz to 0.01 Hz after the cell cycling step.

A Li|separator|LiFePO_4_ cell was assembled to test the electrochemical properties using a Land battery testing system (CT2001A, LAND Electronics). The cathode was prepared by mixing the LiFePO_4_ powder, PVDF and acetylene black at a weight ratio of 8 : 1 : 1 in an NMP solvent. The cathode material was spread on the surface of aluminum foil and dried in an air drying oven at 90 °C for 1.5 h and then in a vacuum drying oven at 120 °C for 4 h. The loading of LiFePO_4_ was about 4.0 mg cm^−2^. The specific loading values of each cell are given in Table S2.† A lithium plate with a thickness of 350 μm and a diameter of 14 mm was used as the anode. The cathodes and separators were cut into discs with a diameter of 11 mm and 16 mm, respectively. The discharge current densities were varied from 0.5 to 3 C to test the rate capability in the voltage range between 2.5 V and 3.8 V. Cycling performance was investigated at a voltage in the range between 2.5 V and 3.8 V at a 0.5 C charge and discharge rate.

## 3. Results and discussion

### 3.1. Morphology and structure

**Fig. 2:**
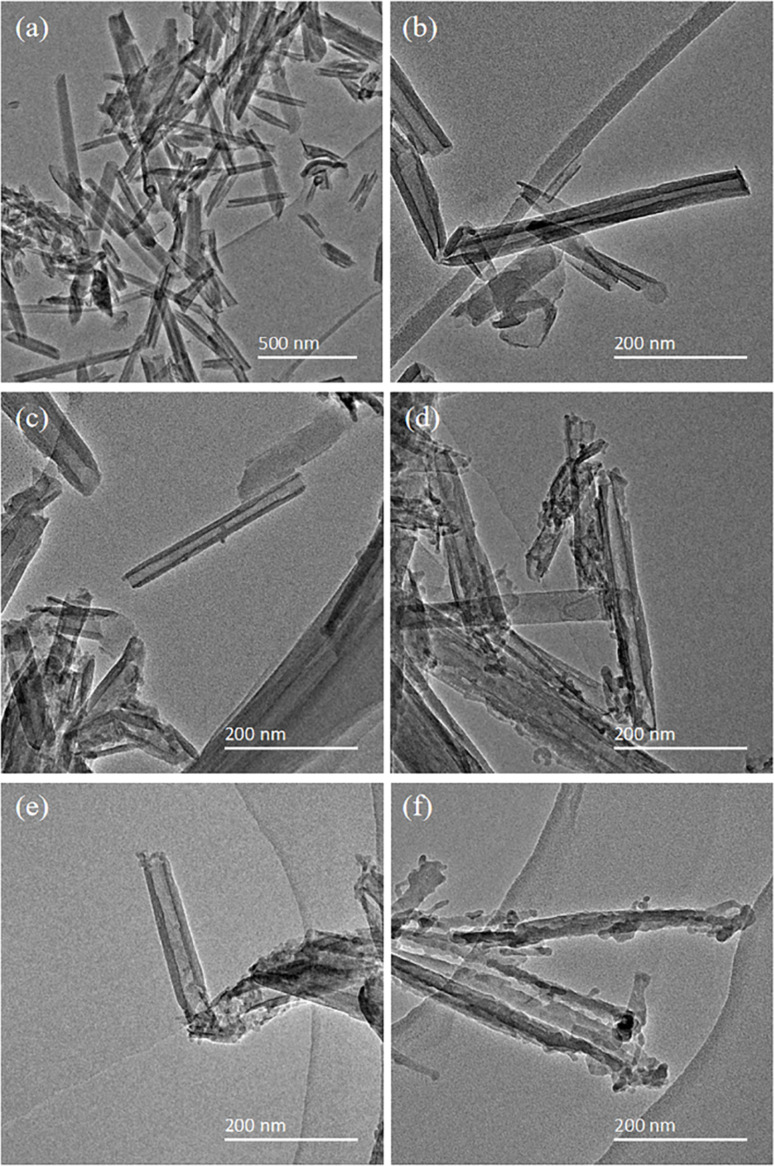
TEM images of (a) and (b) HNTs-0 M, (c) HNTs-0.4 M, (d) HNTs-0.8 M, (e) HNTs-1.2 M and (f) HNTs-4 M.

The structure of HNTs was observed by TEM, and is shown in Fig. 2. Fig. 2(a) and (b) shows the HNTs-0 M exhibiting a typical hollow tube structure, which provided channels for the transport of lithium ions. The inner and outer surfaces of the tubes were flat and smooth. As illustrated in Fig. 2(c–f), with the increase in HCl concentration, the tube walls of HNTs became thinner and the inner walls of the tubes became uneven, indicating that HCl first reacted with the alumina layer on the inner tube walls. Among them, the HNTs-1.2 M had the largest inner diameter, as shown in Fig. 2(e), which was beneficial to the transport of lithium ions by providing more sufficient channels, thereby improving the physical and electrochemical properties of the separator. When the HCl concentration was 4 M, the tube walls were perforated and some nanotubes turned into nanorods made of SiO_2_ (Fig. 2(f)). This was mainly because HCl continuously destroyed the structure of HNTs and dissolved the alumina octahedral sheet, which led to the fracture and collapse of the silica tetrahedral layer on the outer walls.^29^ The nanorods of SiO_2_ blocked some pores and reduced the porosity, which adversely affected the transport of lithium ions and the electrochemical performance of the separator.

**Fig. 3:**
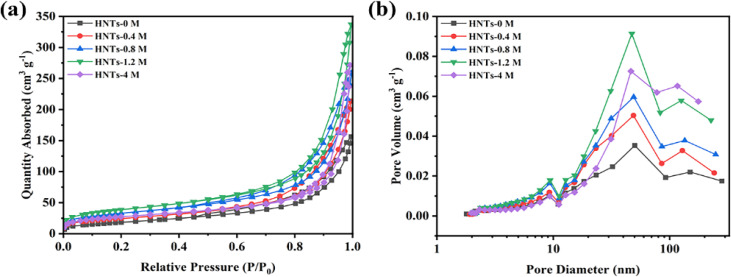
(a) N_2_ absorption and desorption isotherms. (b) Pore size distribution curves of different HNTs.

The pore structure of HNTs before and after HCl treatment was tested by N_2_ absorption and desorption. As shown in Fig. 3(a), all samples showed a type of IV isotherm and H3 hysteresis loop, indicating the mesopore structure of all prepared HNTs. Fig. 3(b) shows the pore size distribution of different HNTs, which are concentrated in mesopores (2–50 nm) and macropores (>50 nm), with a small amount of micropores (<2 nm). The specific surface area and pore volume of different HNTs are listed in Table 1.

**Table 1:** Specific surface area and pore volume of different HNTs

Sample	*S*_BET_ (m^2^ g^−1^)	*V*_total_ (cm^3^ g^−1^)	*V*_mic_ (cm^3^ g^−1^)	*V*_mes_ (cm^3^ g^−1^)	*V*_mac_ (cm^3^ g^−1^)
HNTs-0 M	60.3833	0.2445	0.0027	0.1477	0.0941
HNTs-0.4 M	83.6195	0.3338	0.0008	0.2524	0.0805
HNTs-0.8 M	113.4499	0.4062	0.0015	0.3014	0.1034
HNTs-1.2 M	133.7310	0.5282	0.0000	0.3706	0.1576
HNTs-4 M	95.0716	0.4192	0.0015	0.2331	0.1845

The specific surface area and pore volume of HNTs-0 M were only 60.3833 m^2^ g^−1^ and 0.2445 cm^3^ g^−1^, respectively. With the increase in HCl concentration, the specific surface area and pore volume of HNTs after HCl treatment first increased and then decreased, of which HNTs-1.2 M had the largest specific surface area and the pore volume of 133.7310 m^2^ g^−1^ and 0.5282 cm^3^ g^−1^ respectively. This was consistent with the microstructure changes revealed by TEM images. As the tube walls of HNTs were etched by HCl and the alumina octahedral sheet was dissolved, the formed micropores enlarged the inner diameter of the tube and increased the specific surface area and pore volume, which was conducive to improve the porosity and electrolyte uptake of the separator. However, when the concentration of HCl was too high, the formed nanorods composed of SiO_2_ showed reduced specific surface area and pore volume.

**Fig. 4:**
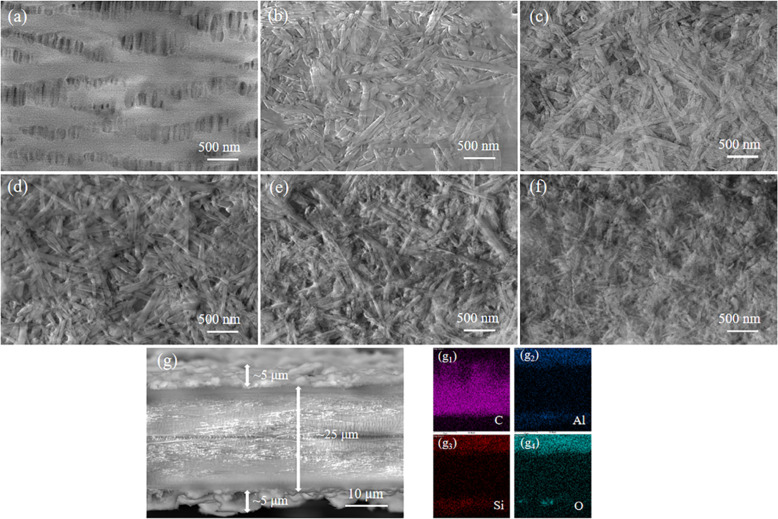
SEM images of the surface of (a) PP, (b) PP/HNTs-0 M, (c) PP/HNTs-0.4 M, (d) PP/HNTs-0.8 M, (e) PP/HNTs-1.2 M and (f) PP/HNTs-4 M. SEM image of the cross-section of (g) PP/HNTs-0 M with the corresponding elemental mappings of (g_1_) C, (g_2_) Al, (g_3_) Si and (g_4_) O.

Fig. 4 depicts the surface morphologies of the PP and PP/HNT composite separators by SEM. Fig. 4(a) shows that the pores of PP separator are rich and evenly distributed. Fig. 4(b–f) shows that the HNTs before and after HCl treatment were coated on the surface of the PP separator, and the three-dimensional pore structure formed by the accumulation of HNTs enabled the separator to absorb and store a large amount of electrolytes. When the concentration of HCl was 4 M, nanorods composed of SiO_2_ were arranged on the surface of the PP separator (Fig. 4(f)). To ensure the uniformity and integrity of the coating, the cross-section of the PP/HNTs-0 M composite separator was scanned (Fig. 4(g)). The distribution of C, Al, Si and O indicated that HNTs were successfully coated on both sides of the PP separator.

### 3.2. FTIR and XRD analysis

**Fig. 5:**
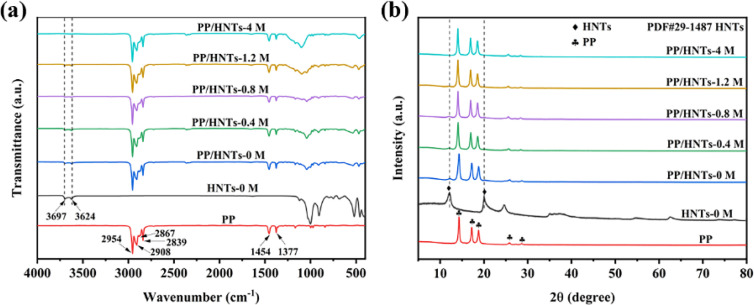
(a) FTIR spectra and (b) XRD patterns of HNTs-0 M, PP and PP/HNT composite separators.

Fig. 5(a) showed the FTIR spectra of HNTs-0 M, PP and PP/HNT composite separators. The spectrum of HNTs-0 M showed two characteristic absorption peaks at 3697 and 3624 cm^−1^, which were attributed to the Al–OH stretching vibration.^30^ For the PP separator, the absorption peaks were observed at 2954, 2908, 2867 and 2839 cm^−1^ (C–H stretching vibration), and 1454 and 1377 cm^−1^ (C–H bending vibration).^31^ The appearance of all the characteristic peaks of HNTs and PP in the FTIR spectra of PP/HNT composite separators indicated the successful combination of PP and HNTs. In addition, due to the loss of alumina octahedral, the strength of the peak at 3697 and 3624 cm^−1^ in the spectra of the PP/HNT composite separators was decreased with the increase in HCl concentration.

The XRD patterns of HNTs-0 M, PP and PP/HNT composite separators are shown in Fig. 5(b). HNTs-0 M had two particularly strong characteristic peaks, which appeared at 2*θ* = 12.1° and 20.0° (JCPD card no. 29-1487).^32^ In the XRD pattern of PP, the characteristic diffraction peaks were at 2*θ* = 14.3°, 17.2° and 18.8°. The characteristic peaks of HNTs and PP appeared simultaneously on the PP/HNT composite separators, indicating that HNTs had been successfully coated on the PP separator. In addition, the strength of the peaks at 2*θ* = 12.1° and 20.0° reduced with the increase in HCl concentration, which indicated that the crystallinity of HNTs reduced gradually due to the loss of alumina octahedral.

### 3.3. Contact angle, electrolyte uptake and porosity

**Fig. 6:**
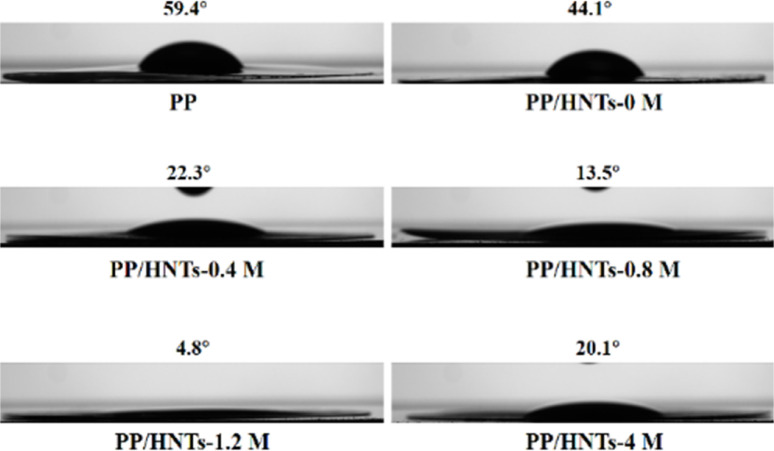
Contact angle photographs of the electrolyte on the PP and PP/HNT composite separators.

Good electrolyte wettability can help the separator to effectively retain the electrolyte during the battery charging and discharging process.^33^Fig. 6 shows the contact angles of different separators to electrolytes. The smaller the contact angle, the better the wettability of the separator to the electrolyte. The contact angle of PP was tested as 59.4°, which meant that it was less wettable to the electrolyte. However, when HNTs treated with HCl of different concentrations were coated on both sides of the PP separator, the contact angles of PP/HNT composite separators were all lower than that of the PP separator. Among them, the contact angle of the PP/HNTs-1.2 M composite separator reached the lowest value of 4.8°, because the HNTs-1.2 M composite separator in the coating layer had the largest specific surface area and pore volume (Table 1). The excellent electrolyte wettability of the PP/HNTs-1.2 M composite separator was expected to enhance the ionic conductivity and make the distribution of the lithium ions uniform, which improved the rate capability and cycling stability of the battery.^34^

To better evaluate the separator properties, the electrolyte uptake and porosity of PP and PP/HNT composite separators are summarized in Table 2. The electrolyte uptake and porosity of the PP separator were 92% and 54%, respectively, which were much lower than those of PP/HNT composite separators. When the HCl concentration was no more than 1.2 M, the electrolyte uptake and porosity gradually increased with the HCl concentration. When the HCl concentration exceeded 1.2 M, the electrolyte uptake and porosity apparently decreased. The result was the same as that of the contact angle, indicating that the PP/HNTs-1.2 M separator had the best affinity for the electrolyte.

**Table 2:** Electrolyte uptake and porosity of PP and PP/HNT composite separators

Samples	Electrolyte uptake (%)	Porosity (%)
PP	92	54
PP/HNTs-0 M	233	69
PP/HNTs-0.4 M	321	71
PP/HNTs-0.8 M	343	74
PP/HNTs-1.2 M	384	79
PP/HNTs-4 M	330	76

### 3.4. Thermal stability

**Fig. 7:**
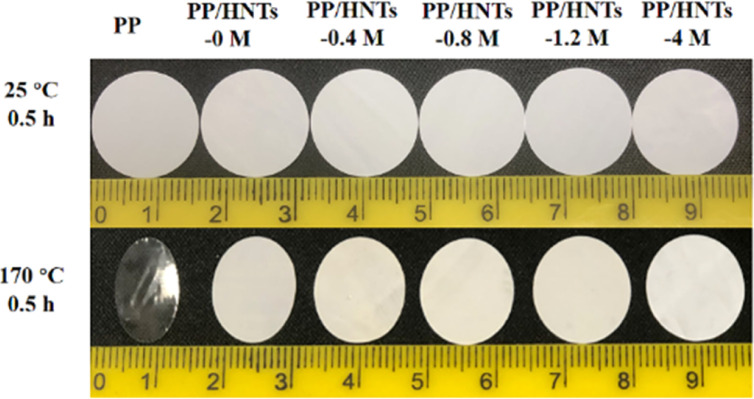
Digital image of the PP and PP/HNT composite separators before and after thermal treatment at 170 °C for 30 min.

The high thermal stability of the separator can avoid the internal short circuit in LIBs. Therefore, its shrinkage at elevated temperatures needs to be tested. The digital image of PP and PP/HNT composite separators before and after thermal treatment at 170 °C for 30 min is shown in Fig. 7. It can be seen that the PP separator experienced severe shrinkage, whereas only a small change was noticed for the PP/HNT composite separators due to the outstanding thermal stability of the HNTs. This implied that the addition of inorganic nanoparticles can effectively improve the thermal stability of the composite separators.

### 3.5. Mechanical properties

**Fig. 8:**
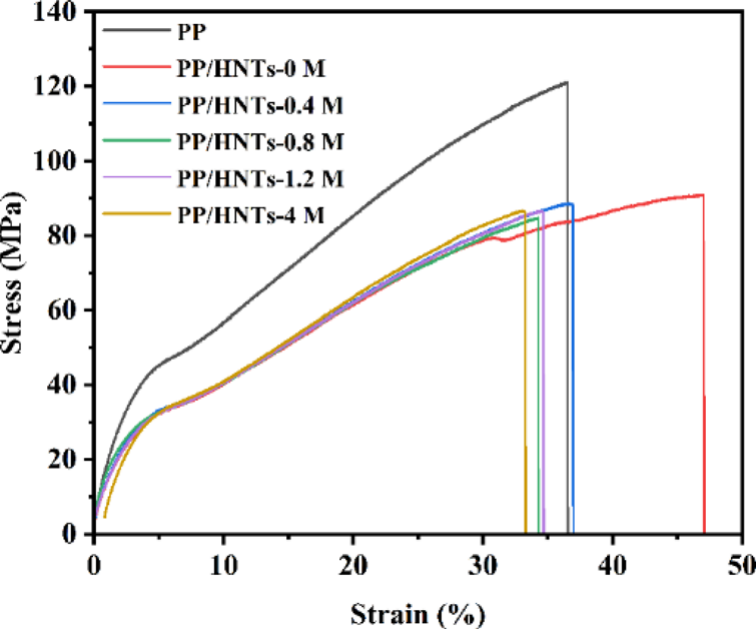
Stress–strain curves of the PP and PP/HNT composite separators.

Excellent mechanical properties of the separator are very important for battery safety. The stress–strain curves of PP and PP/HNT composite separators are shown in Fig. 8. The tensile strength of the PP separator was 121 MPa, which was higher than that of the PP/HNT composite separator. Owing to the small interaction between the particles of the coating, the mechanical properties of the PP/HNT composite separators still depended on the PP separator. In addition, the tensile strength of the PP/HNT composite separators decreased due to the increase in separator thickness.

### 3.6. Electrochemical performances

**Fig. 9:**
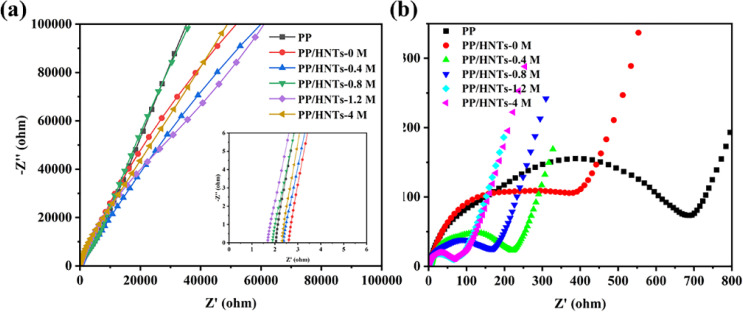
(a) Nyquist plots of SS|separator|SS cells assembled with the PP and PP/HNT composite separators. (b) EIS spectra of the Li/LiFeO_4_ cells assembled with the PP and PP/HNT composite separators after 100 cycles at 0.5 C.

Ionic conductivity is an important parameter that reflects the rate at which lithium ions pass through the separator. The Nyquist plots of PP and PP/HNT composite separators are shown in Fig. 9(a). The intercept of the Nyquist plots on the real *Z* axis at a high frequency represents the bulk resistance (*R*_b_). Table 3 displays the bulk resistance and ionic conductivity of PP and PP/HNT composite separators. Generally, *R*_b_ of the separator is related to its thickness and structure. The increasing thickness increased the *R*_b_ value of the separator.^35^ Hence, with the introduction of the HNT coating layer, the PP/HNTs-0 M, PP/HNTs-0.4 M and PP/HNTs-4 M composite separators had a higher *R*_b_ value than that of the PP separator. By contrast, the microstructure provided by the coating layer can effectively decrease the *R*_b_ value of the separator.^36^ Therefore, the *R*_b_ values of the PP/HNTs-0.8 M and PP/HNTs-1.2 M composite separators were smaller than that of PP, which indicated that the influence of the microstructure of the HNT coating layer on the decrease in the *R*_b_ value is bigger than that of the thickness on the increase in *R*_b_. However, compared to the PP separator (0.61 mS cm^−1^), the PP/HNT composite separators had a higher ionic conductivity due to the higher composite separator thickness and higher electrolyte uptake and porosity. For the PP/HNT composite separators, with the increase in the HCl concentration from 0 M to 1.2 M, the ionic conductivity increases from 0.67 mS cm^−1^ to 1.03 mS cm^−1^, which is consistent with the electrolyte uptake and porosity results. Fig. 9(b) shows the EIS spectra of the Li/LiFeO_4_ cells assembled with PP and PP/HNT composite separators after 100 cycles at 0.5 C. The interfacial resistance of the cell with PP was 698 Ω, whereas the interfacial resistances of the PP/HNT composite separators were all lower than that of PP. Particularly, the interfacial resistance of the cell with PP/HNTs-1.2 M was only 61 Ω, demonstrating that PP/HNTs-1.2 M can effectively improve the stability of LIBs. This result might be attributed to the higher ionic conductivity of PP/HNTs-1.2 M, which facilitated the uniform distribution of the lithium ion and reduced the interface resistance.

**Table 3:** Bulk resistance, ionic conductivity and interfacial resistance of PP and PP/HNT composite separators

Samples	Bulk resistance (Ω)	Ionic conductivity (mS cm^−1^)	Interfacial resistance (Ω)
PP	2.05	0.61	698
PP/HNTs-0 M	2.61	0.67	451
PP/HNTs-0.4 M	2.39	0.73	225
PP/HNTs-0.8 M	1.90	0.92	180
PP/HNTs-1.2 M	1.69	1.03	61
PP/HNTs-4 M	2.32	0.75	62

**Fig. 10:**
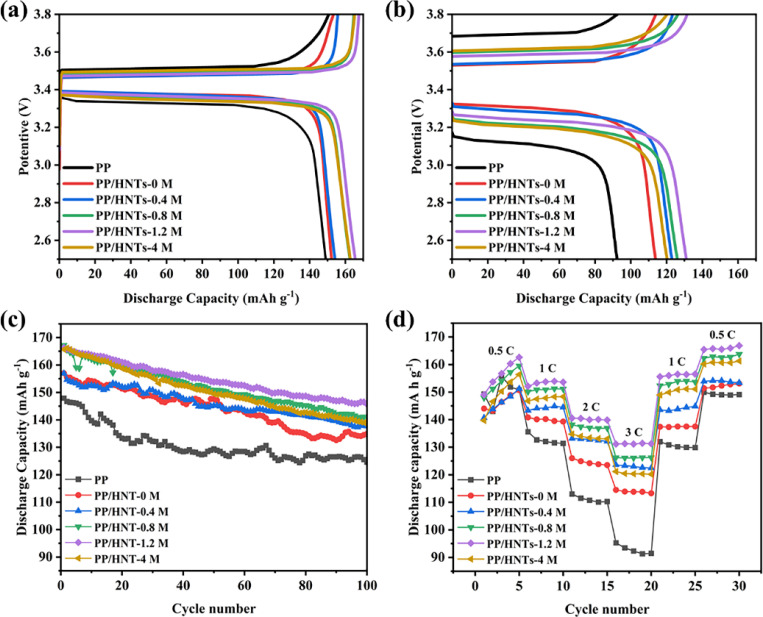
Initial charge/discharge curves of the cells with the PP and PP/HNT composite separators at (a) 0.5 C and (b) 3 C. (c) Cycling performance of the cells with the PP and PP/HNT composite separators at 0.5 C. (d) Rate performance of cells with the PP and PP/HNT composite separators.

Fig. 10(a) shows the initial charge–discharge curves at 0.5 C in the voltage range of 2.5–3.8 V. The discharge capacity of the cells assembled with PP, PP/HNTs-0 M, PP/HNTs-0.4 M, PP/HNTs-0.8 M, PP/HNTs-1.2 M and PP/HNTs-4 M composite separators was 149, 152, 154, 163, 166 and 163 mA h g^−1^, respectively. In Fig. 10(b), for 3 C, the discharge capacity was 92, 114, 123, 126, 131 and 120 mA h g^−1^, respectively. Compared with the PP separator, the cells assembled with PP/HNT composite separators exhibited a higher discharge capacity for low (0.5 C) and high (3 C) rates. In particular, the cells assembled with the PP/HNTs-1.2 M composite separator delivered discharge capacities of 166 mA h g^−1^ (0.5 C) and 131 mA h g^−1^ (3 C). This is due to the higher ionic conductivity, electrolyte uptake and porosity of PP/HNTs-1.2 M.

Fig. 10(c) shows the cycling performance of the assembled cells containing different separators at 0.5 C rate for 100 cycles. The cells with the PP/HNTs-1.2 M composite separator had a higher discharge capacity and the largest capacity retention ratio of 87.6%, while the capacity retention ratios of the PP and PP/HNTs-0 M composite separator were 84.2% and 85.9%, respectively. This result might be attributed to the higher electrolyte uptake and ionic conductivity of the PP/HNTs-1.2 M composite separator, which ensured sufficient participation of lithium ions in redox reactions during cycling.^37^

The rate performance comparison of cells with different separators at 0.5, 1, 2, 3, 1 and 0.5 C is shown in Fig. 10(d). For all separators, with the increase in the rate, the discharge capacity of the cells was reduced. However, no matter at which rate, the cells assembled with the PP/HNTs-1.2 M composite separator had the highest discharge capacity. In contrast, the cell with the PP/HNTs-1.2 M composite separator was still intact at 79.2% of its initial capacity at a rate of 3 C, which was significantly higher than that of PP (61.9%). The excellent performance of the PP/HNTs-1.2 M composite separator was attributed to the high ionic conductivity.

## 4. Conclusions

In summary, we have developed PP/HNT composite separators by coating HNTs treated with hydrochloric acid (HCl) of different concentrations on both sides of PP separators. The effect of HNTs treated with HCl of different concentrations was reflected on the electrolyte uptake, porosity, ionic conductivity and electrochemical performance of the PP/HNT composite separators. The PP/HNT composite separators showed higher electrolyte uptake, porosity and ionic conductivity than those of the PP separator. Moreover, the PP/HNT composite separators exhibited excellent cycling and rate performance in Li/LiFeO_4_ cells. Particularly, the PP/HNTs-1.2 M composite separator showed the highest electrolyte uptake (384%), porosity (79%) and ionic conductivity (1.03 mS cm^−1^). In addition, the cells assembled with the PP/HNTs-1.2 M composite separator exhibited the highest initial discharge capacity (166 mA h g^−1^ at 0.5 C and 131 mA h g^−1^ at 3 C) and the largest capacity retention ratio (87.6%) at 0.5 C for 100 cycles. Therefore, the PP/HNTs-1.2 M composite separator is a good candidate for high-performance lithium-ion batteries.

## Author contributions

Qinting Deng: methodology, data curation, investigation, writing – original draft. Xiuyun Chuan: writing – review & editing, project administration, supervision. Fangfang Liu: formal analysis, writing – review & editing. Yupeng Zhao: methodology, conceptualization. Shizhi Huang: methodology, conceptualization. Jianyang Wu: resources.

## Conflicts of interest

The authors declare no conflicts of interest.

## Supplementary Material

RA-014-D4RA02164A-s001
