# Supplementary material for: Development of composite separators by coating hydrochloric acid-treated halloysite nanotubes on polypropylene separators for lithium-ion batteries
Source: RSC Adv. 2024 May 24;14(24):16912–20. doi: 10.1039/d4ra02164a (PMC11123604; doi:10.1039/d4ra02164a)
Supplement: RA-014-D4RA02164A-s001 [file RA-014-D4RA02164A-s001.pdf]

Electronic Supplementary Material (ESI) for RSC Advances.
This journal is © The Royal Society of Chemistry 2024

Table S1 The mass of HNTs before and after HCl treatment.

HCl concentration (M)	Mass before HCl treatment (g)	Mass after HCl treatment (g)	Mass reduction rate (%)
0.4	1.30	1.13	13%
0.8	1.30	0.99	24%
1.2	1.30	0.83	36%
4	1.30	0.60	54%
6	1.30	0.60	54%
8	1.30	0.60	54%

Table S2 The loading of $LiFePO_4$ in each cell assembled with PP and PP/HNTs composite separators.

Samples	Loading in rate performance (mg cm^{-2})	Loading in cycling performance (mg cm^{-2})
PP	3.91	3.96
PP/HNTs-0 M	3.91	4.20
PP/HNTs-0.4 M	4.65	4.45
PP/HNTs-0.8 M	3.85	3.84
PP/HNTs-1.2 M	3.72	3.86
PP/HNTs-4 M	3.84	3.65
